# Methionine Sulfoxide Reductase B1 Regulates Hepatocellular Carcinoma Cell Proliferation and Invasion via the Mitogen-Activated Protein Kinase Pathway and Epithelial-Mesenchymal Transition

**DOI:** 10.1155/2018/5287971

**Published:** 2018-05-10

**Authors:** Qiang He, Hui Li, Fanzhi Meng, Xiangjun Sun, Xu Feng, Jiang Chen, Libo Li, Jinghua Liu

**Affiliations:** ^1^Department of Hepatobiliary Surgery, Linyi People's Hospital, 27th of East Jiefang Road, Linyi, Shandong 276000, China; ^2^Human Resource Department, Linyi People's Hospital, 27th of East Jiefang Road, Linyi, Shandong 276000, China; ^3^Department of General Surgery, Sir Run Run Shaw Hospital, Zhejiang University School of Medicine, Hangzhou, Zhejiang 310016, China

## Abstract

Methionine sulfoxide reductase B1 (MsrB1) is a member of the selenoprotein family, which contributes to the reduction of methionine sulfoxides produced from reactive oxygen species (ROS) by redox processes in energy pathways. However, few studies have examined the role of MsrB1 in human hepatocellular carcinoma (HCC). We observed that MsrB1 is highly expressed in HCC tissues and that its expression correlated with the prognoses of patients with HCC after hepatectomy. *In vitro*, knockdown of MsrB1 inhibits HCC cell growth by MTT and EdU proliferation assay, and MsrB1 interference enhances H_2_O_2_/trx-induced apoptosis. We observed that phosphorylation of the key proteins of the MAPK pathway, namely, ERK, MEK, and p53, was inhibited, but PARP and caspase 3 were increased, thus infecting mitochondrial integrity. *In vivo*, MsrB1 knockdown effectively inhibited tumor growth. Furthermore, MsrB1 knockdown reduced HCC cell migration and invasion in a transwell assay through inhibition of cytoskeletal rearrangement and spread. This change was linked to epithelial-mesenchymal transition (EMT) inhibition resulting from increases in E-cadherin expression and decreases in expression in TGF-*β*1, Slug, MMP-2/9, and so on. MsrB1 regulates HCC cell proliferation and migration by modulating the MAPK pathway and EMT. Thus, MsrB1 may be a novel therapeutic target with respect to the treatment of HCC.

## 1. Introduction

Hepatocellular carcinoma (HCC) is the fifth most common cancer worldwide and causes half a million deaths each year. In addition to novel therapeutic methods, new and useful prognosis-determining methods, especially methods enabling clinicians to monitor biotherapies, would be extremely beneficial with respect to the treatment of this disease. Among the antioxidant enzymes induced by reactive oxygen species (ROS), methionine sulfoxide reductases (MSRs) are unique in their ability to direct protein repair and indirectly scavenge ROS. Within this subfamily, MsrB1, a selenoprotein that contains a selenocysteine residue in place of the catalytic cysteine residue normally present in other MsrBs [1, 2], displays high catalytic activity toward protein-based R-Met(O) and low efficient activity toward free Met(O) [2].

In this study, we observed that MsrB1 is highly expressed in HCC tissues and that its expression correlated with the prognoses of patients with HCC after hepatectomy. MsrB1 interfered with HCC cell proliferation and invasion *in vitro*/*in vivo*. This change was linked to related processes of the mitogen-activated protein kinase (MAPK) pathway and epithelial-mesenchymal transition (EMT). MsrB1 knockdown in HCC cells resulted in proliferation and metastasis downregulation, suggesting that this factor may be suitable as a biological target for tumor therapy. Targeting antioxidant defense mechanisms may be a suitable strategy for specifically killing HCC cells while sparing normal cells [3]. Therefore, in addition to oncogenes and tumor suppressors, MsrB1 may be the next important targets for future anticancer drug discovery studies.

## 2. Materials and Methods

### 2.1. Clinical Specimen Collection

Samples from 135 patients who underwent hepatic resection in our hospital (Sir Run Run Shaw Hospital, Zhejiang University School of Medicine, Zhejiang, China) between January 2006 and December 2012 were collected for this study. Letters of consent were obtained from all patients, and our experimental protocols were approved by the local ethics committee. Patient charts were reviewed to obtain clinical data regarding age, gender, tumor size, AFP levels, HBsAg positivity, portal vein-emboli and metastases, TNM stage (AJCC), and tumor differentiation.

### 2.2. Cell Culture

Seven human HCC cell lines (HepG2, Hep3B, Huh-7, Bel-7402, SK-Hep-1, SMMC-7721, and MHCC-LM3) and a liver cell line (HL7702) were purchased from Cell Bank of Type Culture Collection of Chinese Academy of Sciences, Shanghai Institute of Cell Biology, Chinese Academy of Sciences, and were cultivated in accordance with the supplier's instructions. The HepG2, Hep3B, Huh-7, SK-Hep-1, and HCCLM3 cell lines were cultured in Dulbecco's modified Eagle medium (DMEM; Gibco-Invitrogen, Carlsbad, CA, USA) supplemented with 10% fetal bovine serum (FBS), and the snu387, Bel-7402, SMMC-7721, and HL7702 cell lines were cultured in 1640 complete medium supplemented with 10% FBS.

### 2.3. Antibodies

Antibodies to phosphorylated Erk1/2 (#4376, diluted 1/1000), Mek (#9154, diluted 1/1000), p53 (#2521, diluted 1/1000), cleaved PARP (#9542, diluted 1/1000) and caspase 3 (#9661, diluted 1/1000), PCNA (#2586, diluted 1/1000), and EMT Antibody Sampler Kit (#9782, diluted 1/500–1000) were all purchased from Cell Signaling Technology Inc. Antibodies to MsrB1 (ab71175, diluted 1/1000), ki67 (ab209897, diluted 1/1000), and TGF-*β*1 (ab155264, diluted 1/500) were all purchased from Abcam. E-cadherin (sc71008, diluted 1/1000) was obtained from Santa Cruz (USA), and *β*-actin was obtained from Epitomics, an Abcam company (Cambridge, MA, USA).

### 2.4. MsrB1 Expression in Tissues and Cells

We selected tissues from 15 tumor-free liver samples and 7 HCC samples, as well as HCC cell lines (HePG2, HeP3B, LM3, and BEL-7402) and a liver cell line (HL-7702), for semiquantitative RT-PCR amplification of specific genes. Then, we performed quantitative real-time PCR (qPCR) amplification of MsrB1 to detect its mRNA expression levels in HL-7702, HePG2, HeP3B, and BEL-7402/4/5 cells. The MsrB1 antibody was used to detect the MsrB1 protein expression in 8 different HCC cell lines (Huh7, HepG2, Hep3B, LM3, BEL-7402, SMMC-7721, snu387, and SK-HeP1). This procedure has been described in detail in a subsequent section.

### 2.5. MsrB1 TaqMan Copy Number Assay

To evaluate relative DNA copy numbers, we used 56 pairs of DNA samples from the cancer tissues and normal tissues of patients with HCC. qPCR amplification was conducted using a TaqMan Copy Number Assay Kit (Hs03918287_cn; ABS, USA). The PCR reactions and analyses were performed according to the manufacturer's instructions. Briefly, PCR was conducted with a total sample volume of 20 *μ*l, which contained template DNA (20 ng), and the threshold cycle number was determined using SDS Software v 1.X. A TaqMan copy number assay was performed to detect the MsrB1 gene, and a reference assay was performed to detect both copies of the human RNase P H1 RNA gene in a diploid genome. The reactions were run simultaneously on an Applied Biosystems Real-Time PCR System, and Applied Biosystems CopyCaller software was used for post-PCR data analysis of the copy number quantitation experiment results.

### 2.6. MsrB1 Expression and HCC Patient Prognosis

We downloaded and analyzed the RNA-seq data pertaining to 178 HCC cases from the TCGA database. Based on MsrB1 expression levels, we classified the data into 2 groups separated by a median value and analyzed the correlation between MsrB1 mRNA expression and patient prognosis.

A total of 135 pathological sections were immunohistochemically stained, and 125 of these cases underwent complete follow-up evaluations. Immunohistochemistry (IHC) was performed as described previously [4].

Tissue sections were incubated with an MsrB1 primary antibody (1 : 100 dilution; DMAB14855, Reactive Diagnostics, USA) overnight at 4°C. Then, the appropriate secondary antibody was applied to the sections, as was a diaminobenzidine (DAB)+ chromogen. The tissue slides were lightly counterstained with hematoxylin and sealed. MsrB1 expression was scored based on the numbers of positive cells and the intensity of cytoplasmic staining using the following four-point system: −, negative; +, weak; ++, moderate; and +++, strong. To examine the association between MsrB1 expression and clinicopathological features, we divided the patients into the following two groups: a low expression (−/+) group and a high expression (++/+++) group. The immunostaining results were scored independently by two pathologists blinded to patient clinical information.

### 2.7. MsrB1-Knockdown Lentiviruses and Stable Infection of HCC Cell Lines

The HCC cell lines LM3 and snu387 were infected with MsrB1-knockdown shRNA or negative-control shRNA with puromycin. Stably infected cells were selected for further study.

### 2.8. Gene Expression Profiling

Total RNA was extracted from the snu387 cells after MsrB1 shRNA transfection. Three biological replicates were used. Gene expression profiling was conducted by the Biotechnology Corporation (Shanghai, China) using Affymetrix PrimeView human gene expression arrays. All data were analyzed according to the manufacturer's protocol. Raw data generated from Affymetrix CEL files were normalized by RMA background correction, and values were log2 transformed. To enrich the *P* values of each GO and KEGG term, we performed Fisher's exact test to calculate the *P* values. R package stats were used to calculate the FDRs (*q* values) using the BH method.

### 2.9. Cell Viability Assay

Cell viability was determined via MTT assay. Briefly, negative-control (NC) and knockdown (sh-MsrB1) cells were seeded in 96-well flat-bottomed plates at a density of 1 × 10^4^ cells/well. After 24 h, the medium was replaced with medium with/without sorafenib (3 *μ*g/ml) and incubated for 24, 48, or 72 h at 37°C and 5% CO_2_. The absorbance was measured on a microplate reader (Thermo Fisher Scientific, USA) at 490 nm.

Then, we seeded cells and wild-type cells in 6-well plates at a 1 : 1 ratio. After incubation, flow cytometry was used to measure the percentage of green fluorescence.

### 2.10. EdU Proliferation Assay

The effects of MsrB1 on HCC cell proliferation were also tested using 5-ethynyl-2′-deoxyuridine (EdU) assay. Transfected cells (1 × 10^4^ cells/well) were exposed to 50 *μ*M EdU solution (RiboBio, Guangzhou, China) in 6-well plates for 2 h at 37°C. The cells were fixed with 4% formaldehyde for 30 min at room temperature and treated with 0.5% Triton X-100 for 10 min for permeabilization. After being washed thrice with PBS, the cells were incubated with an Apollo reaction cocktail (500 *μ*l/well) for 30 min in the dark. The DNA was stained with Hoechst 33342 (100 *μ*l/well) for 20 min and visualized using fluorescence microscopy.

### 2.11. Colony Formation Assays

For colony formation assays in 2D culture, we separately plated 1000 cells from the indicated two groups in 10 cm^2^ dishes and incubated the cells for 2 weeks at 37°C and 5% CO_2_. The surviving colonies (≥50 cells/colony) were quantified after crystal violet staining.

### 2.12. *In Vivo* Subcutaneous Tumor Model

All *in vivo* experimental protocols were approved by the appropriate ethics committee and the review board of Sir Run Run Shaw Hospital and were conducted in accordance with national guidelines. Viable LM3 cells (3.5 × 10^6^ cells in 0.1 ml of PBS) were subcutaneously injected into the right dorsal flank of 5-week-old female BALB/c nude mice (8 mice per group). Tumor volume was assessed every 2 days for 8 weeks and was calculated using the following formula: ((short diameter)^2^ × (long diameter))/2. The MsrB1 antibody was used to detect the expression of MsrB1 in tumors of both groups of mice.

### 2.13. Cell Cycle and Apoptosis

Cell cycle distributions and apoptotic cell percentages were determined by flow cytometry, as described previously [5].

### 2.14. Mitochondrial Cell Immunofluorescence

The treated cells were cultured on glass coverslips and fixed in 4% paraformaldehyde in PBS for 10 min, permeabilized in 0.1% Triton X-100 in PBS for 4 min, blocked with 1% BSA/PBS for 1 h, and then incubated with Mito-Tracker Green (Beyotime, Nanjing, China) for 1 h at room temperature. The cell nuclei were counterstained with Hoechst 33342, and images were acquired using a fluorescence microscope.

### 2.15. Cell Migration Assay

The cells were trypsinized and resuspended in DMEM containing 1% FBS at a density of 1 × 10^6^ cells/ml. Part of the cell suspension (100 *μ*l) was added to the upper transwell chamber (Corning, Corning, NY, USA), and DMEM (600 *μ*l) containing 2.5% FBS was added to the lower chamber. After the LM3 cells had incubated for 24 h and the snu387 cells had incubated for 12 h, the cells that remained in the upper chamber were carefully removed. The side facing the lower chamber was stained with DAPI, and the attached cells were counted under a fluorescence microscope. Then, the cells were washed with glacial acetic acid to measure the absorbance at 490 nm.

### 2.16. Cell Immunofluorescence for Cytoskeletal Evaluation

The cells were cultured on glass coverslips and fixed in 4% paraformaldehyde in PBS for 10 min, permeabilized in 0.1% Triton X-100 in PBS for 4 min, blocked with 1% BSA/PBS for 1 h, and then incubated with rhodamine-conjugated phalloidin (Invitrogen, CA, USA) diluted 1 : 100 in a blocking solution for 1 h at room temperature. The cell nuclei were counterstained with DAPI, and images were acquired using a fluorescence microscope.

### 2.17. RNA Extraction, Reverse Transcription, and Real-Time qPCR

Total RNA was extracted from cultured cells using an Ultrapure RNA Extract Kit (CWBiotech, Beijing, China), and reverse transcription was performed with 1 *μ*g of total RNA using an iScript cDNA Kit (Bio-Rad, CA, USA) with random hexamers. Real-time qPCR was performed using an ABS-7500 Real-Time PCR System (Invitrogen Life Technologies), and SYBR EvaGreen mixed with low ROX (Bio-Rad) was used for product detection.

Complementary DNA was amplified for MsrB1 detection using 5′-*AGCCGCTCGAAGTATGCAC*-3′ as the forward primer and 3′-*CTTGCCACAGGACACCTTCA*-5′ as the reverse primer. To normalize gene expression data, we used *β*-actin as a reference gene and amplified it using 5′-*CATGTACGTTGCTATCCAGGC*-3′ as the forward primer and 3′-*CTCCTTAATGTCACGCACGAT*-5′ as the reverse primer. All of the primers used for the above experiments are shown in Table 1. The following cycling program conditions were used: 5 min at 95°C, followed by 40 cycles at 95°C for 15 s and 60°C for 30 s.

### 2.18. Western Blot Analysis

We used antibodies to detect target protein expression (see antibody above) in the LM3 and snu387 cell lines.

Western blotting was performed as follows: the transfected cell proteins were collected and stored at −80°C after being centrifuged at 12,000*g* for 15 min. Protein content was determined using bicinchoninic acid assay (BCA, Thermo Fisher). After denaturation, the proteins were separated by gel electrophoresis using 8–12% SDS-PAGE and transferred to a PVDF membrane for 1-2 hours for blocking using 5% skimmed milk. The membrane was subsequently washed with TBST and incubated with the appropriate antibodies overnight at 4°C before being washed three times with TBST and incubated with the indicated secondary antibody (goat anti-rabbit/mouse IgG 1 : 1000) for 2 h at room temperature. The membrane was then rewashed with TBST before being treated with ECL liquid and placed in a darkroom to allow the reaction to run to completion. *β*-Actin was used as a positive control.

### 2.19. Statistical Analyses

Statistical analysis was performed using SPSS 17.0, and results were expressed as the mean ± standard deviation (SD). Analysis of variance was used to analyze variance among all the groups, and the potential associations between MsrB1 gene expression and clinicopathological parameters were evaluated using chi-square tests or Fisher's exact test. Overall survival rates were calculated using the Kaplan-Meier method, and the significance of the differences between survival curves was assessed using the log-rank test. We performed independent sample *t*-tests, and *P* < 0.05 was considered statistically significant.

## 3. Results

### 3.1. Upregulation of MsrB1 in HCC Is Correlated with Poor Prognosis

**Figure 1:**
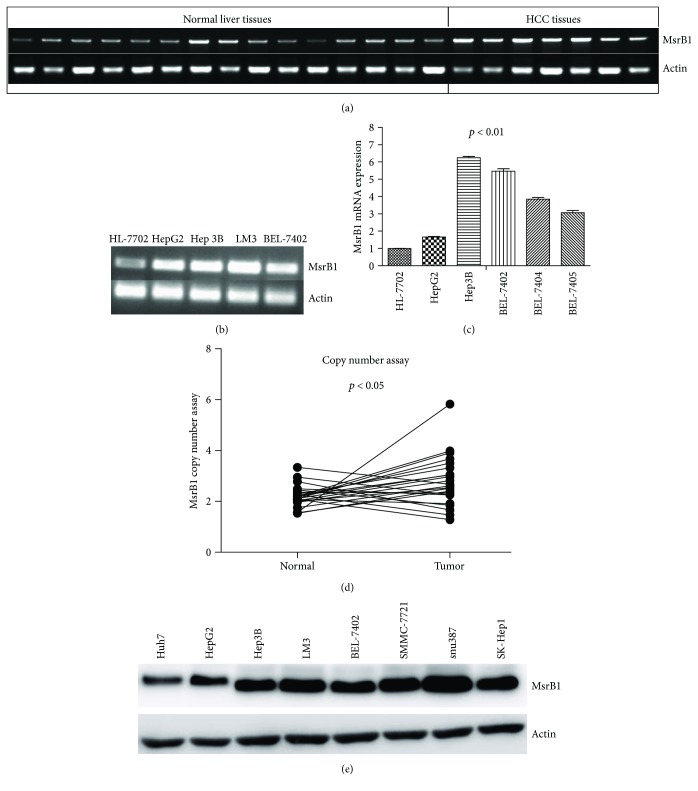
Expression of MsrB1 in tissues and cell lines of HCC. (a) MsrB1 mRNA expression was elevated in 6 HCC tumors than in 9 normal liver tissues detected by RT-PCR. (b) MsrB1 mRNA expression was increased in HepG2, Hep3B, LM3, and BEL-7402 cell lines than in HL-7702 detected by RT-PCR. (c) MsrB1 mRNA expression was increased in HepG2, Hep3B, BEL-7402, BEL-7404, and BEL-7405 cell lines than in HL-7702 detected by q-PCR (*P* < 0.01). (d) The DNA copy number assay showed that the MsrB1 copy number was stepped up in HCC tissues than in adjacent normal tissues. (e) The MsrB1 expression level was increased in HepG2, Hep3B, LM3, snu387, SK-hep1, and BEL-7402 and decreased in Huh7 and SMMC-7721 detected by Western blotting.

To detect MsrB1 expression in HCC tissues and paratumor tissues, we analyzed MsrB1 mRNA levels in tissue samples from 9 patients with tumor-free liver disease and 6 patients with HCC using RT-PCR. We found that MsrB1 mRNA expression was upregulated in 5 of the 6 HCC tissue samples compared with 8 of the 9 tumor-free liver disease tissue samples (Figure 1(a)). We also selected 8 HCC cell lines and a liver cell line, HL-7702, to evaluate MsrB1 expression using RT-PCR, qPCR, and Western blotting (Figures 1(b), 1(c), and 1(e), resp.), which produced results consistent with those of the above human tissue sample analyses. We performed TaqMan copy number assay to determine the differences in copy number between 23 samples of DNA from normal tissues and tumor tissues in patients with HCC. The results of our analysis showed that MsrB1 DNA copy number in the cancer tissue samples was twice as high as that in the paratumor tissue samples (Figure 1(d)).

**Figure 2:**
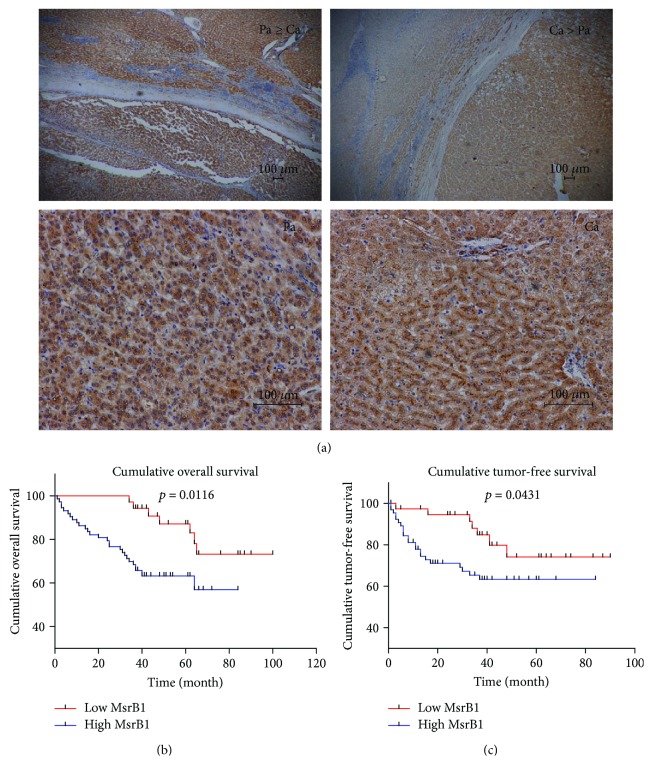
Tissue expression of MsrB1 in HCC and its relation to survival in survival analysis. (a) MsrB1 expression level is upregulated in carcinoma (Ca) and/or paratumor (Pa) tissue. (b) Compared with patients with low expression, patients with high MsrB1 expression had worse overall survival (*P* = 0.0116). (c) Compared with patients with low expression, patients with high MsrB1 expression had worse tumor-free survival (*P* = 0.0431).

According to the results of a prognosis analysis based on IHC staining (Figure 2(a)), MsrB1 was positively expressed in 84/135 patients, and high MsrB1 expression was correlated with poor prognoses with respect to overall survival and tumor-free survival (*P* < 0.05; Figures 2(b) and 2(c), resp.). Compared with patients with low expression, patients with high MsrB1 expression had worse overall survival and tumor-free survival. The 1-, 3-, and 5-year survival rates were much worse in the high group than in the low group. The 5-year survival rate is 64.3% in high-expression patients versus 82.7% in the low group. The 3-year tumor-free survival is 67.6% in the high group compared to 81.4% in the low group. The survival trend was more noticeable via TCGA database analysis than via mRNA analysis (*P* = 0.201; Figure S1).

The results of the IHC analysis of the relationship between MsrB1 expression levels and clinicopathological characteristics showed that MsrB1 expression was correlated with tumor size (*P* = 0.04, Table 2), age (*P* = 0.028, Table 2), liver cirrhosis (*P* = 0.04, Table 2), and Barcelona Clinic Liver Cancer (BCLC) stage (*P* = 0.03, Table 2).

### 3.2. ShRNA Knockdown of MsrB1 Expression in HCC Cells and Enrichment Analysis

We examined MsrB1 expression HCC cell lines and selected LM3 and snu387 cells as target cells to proceed with the latter experiments by qPCR and Western blotting. MsrB1 expression was silenced due to shRN-MsrB1 interference. MsrB1 expression was reduced at the protein level (Figure S2A).

MsrB1 mRNA sequence expression levels were measured in sh-MsrB1 and sh-NC snu387 cells (Figures S2A and S2B, resp.). We then evaluated the associations between these expression levels and various cellular processes via GO and KEGG enrichment analysis. GO enrichment analysis showed that G-protein-coupled activity, G-protein-coupled receptor signaling pathways, the cytoplasm, DNA replication, positive regulation of apoptotic processes, extracellular matrix organization, and positive regulation of cell proliferation were the main cellular processes associated with MsrB1 expression (Figure S2C), while KEGG enrichment analysis showed that DNA replication, cell cycle, and the p53 signaling pathway were the 3 major cell events associated with MsrB1 expression (*P* < 0.05; Figure S2D).

### 3.3. Knockdown of MsrB1 Inhibits Cell Growth in HCC Cells

**Figure 3:**
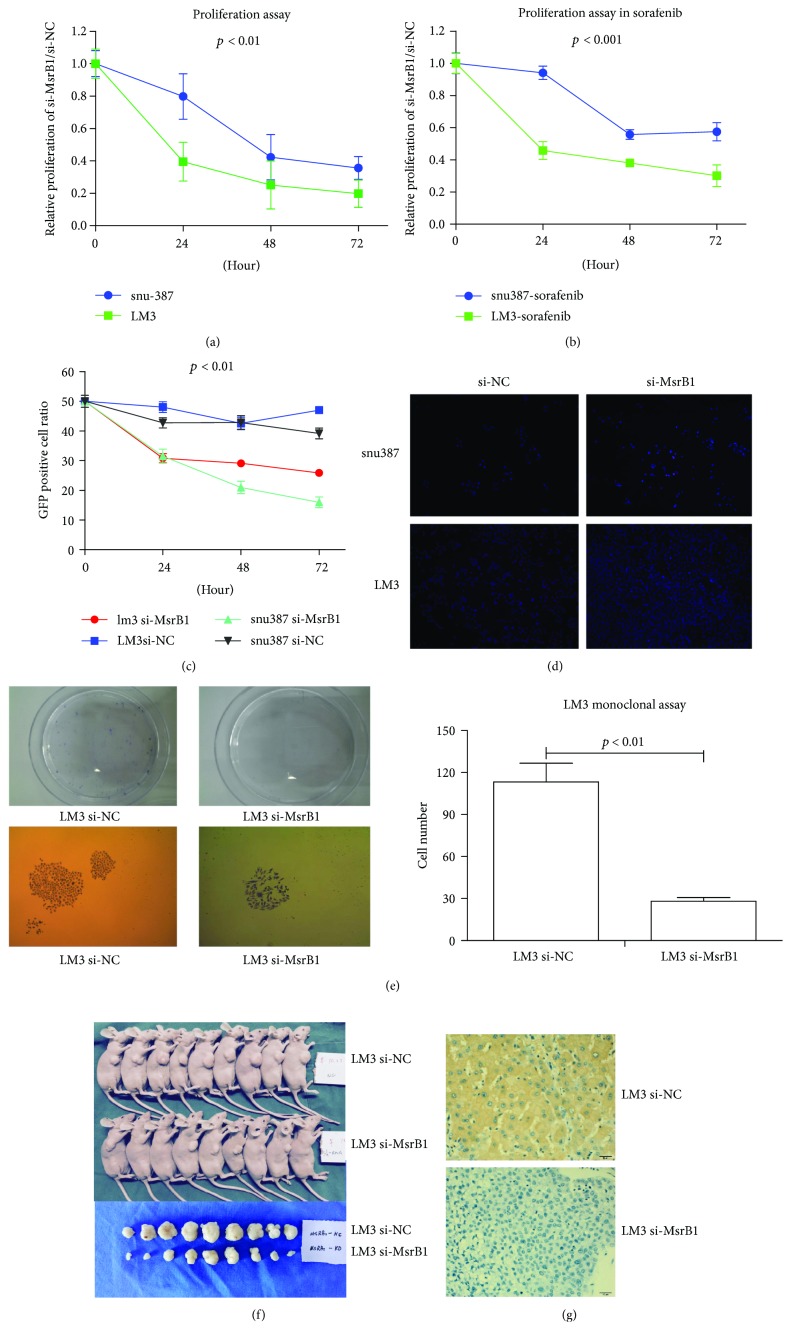
MsrB1 inhibits HCC cell proliferation *in vitro* and *in vivo*. (a) The MTT assay showed that MsrB1 knockdown inhibits the proliferation of LM3 and snu387 cells in 24, 48, and 72 h (*P* < 0.01). (b) The MTT assay showed that MsrB1 knockdown inhibits the proliferation of LM3 and snu387 cells in 24, 48, and 72 h under stress of sorafenib (*P* < 0.001). (c) Flow cytometry showed that MsrB1 knockdown inhibits the proliferation rate of HCC cells. (d) The EdU assay showed that the proliferation percentage of red-fluorescent cells was lower than that of control cells. (e) MsrB1 knockdown inhibits the colony formation of HCC cells. (f) MsrB1 knockdown inhibits the tumor volumes of the subcutaneous tumor of the two groups in 7 weeks in LM3 cells. (g) The expression of MsrB1 in the tumors of both groups of mice was shown to be much lower in the sh-MsrB1 group than in the sh-NC group.

Stably infected LM3 and snu387 cells were cultured for 24 h, 48 h, and 72 h, and cell viability was measured using MTT assay (Figure 3(a)). The cell viability percentages (sh-MsrB1 versus sh-NC) were 39.41%, 25.07%, and 19.71% in LM3 cells and 79.78%, 42.26%, and 35.54% in snu387 cells after 24 h, 48 h, and 72 h, respectively.

In the sorafenib-treated group, the cell viability percentages were 45.75%, 38.09%, and 30.14% in LM3 cells and 94.05%, 55.72%, and 57.45% in snu387 cells after 24 h, 48 h, and 72 h, respectively (Figure 3(b)). In the group in which sh-MsrB1 and sh-NC cells were cultured with wild-type cells at a 1 : 1 ratio, the GFP percentages were 64.10%, 68.37%, and 54.98% in LM3 cells and 73.66%, 48.85%, and 40.82% in snu387 cells after 24 h, 48 h, and 72 h, respectively (Figure 3(c)).

HCC cell proliferation was again tested using an EdU assay, the results of which showed that the proliferation percentage of red-fluorescent cells (representing proliferation in the MsrB1-interference cell line) was lower than that of control cells (Figure 3(d)). Similarly, the results of the colony formation assay showed that 36 colonies in the sh-MsrB1 group had a cell number ≥ 50 and 144 colonies in the sh-NC group had a cell number ≥ 50 (Figure 3(e)).

### 3.4. *In Vivo* HCC Formation in a Subcutaneous Tumor Model

LM3 cells were used to examine HCC formation in female BALB/c nude mice. After 8 weeks, the sizes of the HCC tumors in the sh-MsrB1 group were significantly smaller than those in the sh-NC group (Figure 3(f)), indicating that MsrB1 knockdown inhibited tumor growth in a xenograft tumorigenicity model. Furthermore, the expression of MsrB1 in tumors of both groups of mice was shown to be much lower in the sh-MsrB1 group than in the sh-NC group (Figure 3(g)).

### 3.5. Knockdown of MsrB1 Enhances H_2_O_2_/trx-Induced Apoptosis in HCC Cells

**Figure 4:**
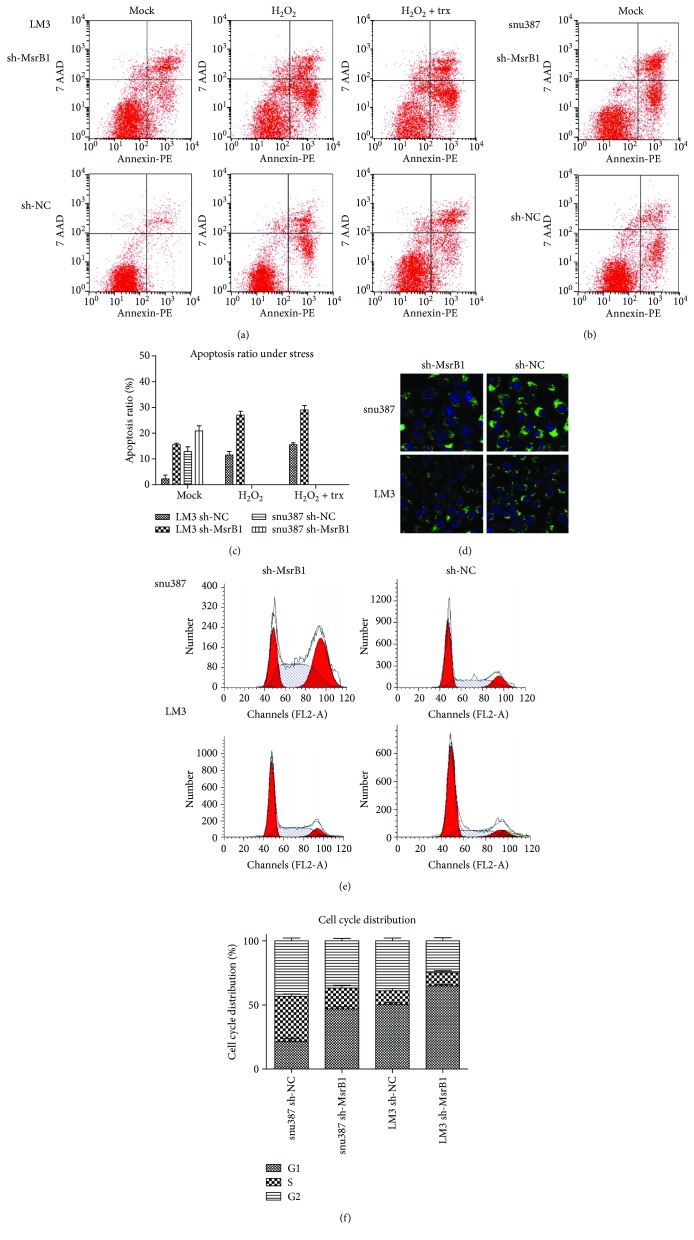
MsrB1 induced apoptosis and cell cycle arrest of HCC cells. (a, c) Knockdown of MsrB1 enhances H_2_O_2_/trx-induced apoptosis of LM3 cells. (b, c) Knockdown of MsrB1 enhances apoptosis of snu387 cells. (d) Mitochondrial integrity completely disappeared in HCC cells due to MsrB1 knockdown, resulting in the fragmentation of some mitochondria. (e, f) Knockdown of MsrB1 induced S/G2 phase arrest and G1 phase decrease.

Using flow cytometry, we evaluated whether MsrB1-interference-induced growth inhibition in HCC cells was related to apoptosis. The rates of apoptosis were 15.58% and 2.27% and 20.9% and 12.9%, in the LM3- and snu387-cell MsrB1-interference and sh-NC groups, respectively (Figures 4(a) and 4(b), resp.). After LM3 cells were treated with H_2_O_2_ and H_2_O_2_/thioredoxin (trx), the apoptosis percentages were 27.05% and 11.57% (H_2_O_2_ group) and 29.08% and 15.58% (H_2_O_2_/trx group) in the MsrB1-interference and control groups, respectively, differences that were statistically significant (Figures 4(a) and 4(c), resp.). These results indicate that MsrB1 knockdown induced apoptosis in HCC cells. Taken together, these findings suggest that MsrB1 protects HCC cells from apoptosis under H_2_O_2_/trx-induced stress.

### 3.6. Knockdown of MsrB1 Induces a Breakdown in Mitochondrial Integrity in HCC Cells

While detecting apoptotic phenomena, we noted visible fluorescence indicative of a breakdown in mitochondrial integrity, especially mitochondrial membrane integrity. Mitochondrial integrity completely disappeared in HCC cells due to MsrB1 knockdown, resulting in the fragmentation of some mitochondria (Figure 4(d)).

### 3.7. Knockdown of MsrB1 Decreases Cell Cycle Progression in HCC Cells

Using flow cytometry, we evaluated whether cell growth inhibition caused by sh-MsrB1 was related to cell cycle arrest. We determined that sh-MsrB1 induced S/G2 phase arrest and resulted in a decreased percentage of cells in the G1 phase (Figures 4(e) and 4(f)).

### 3.8. Knockdown of MsrB1 Inhibits HCC Cell Migration

**Figure 5:**
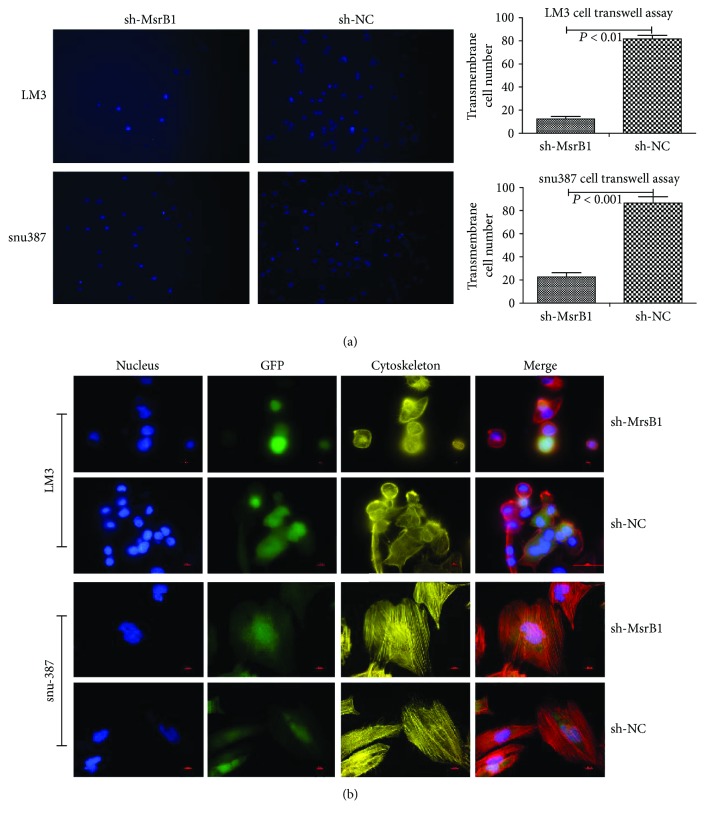
MsrB1 knockdown inhibited the migration of HCC cells. (a) The transwell assay demonstrated that knockdown of MsrB1 reduced the migration of HCC cells. (b) Knockdown of MsrB1 leads to cytoskeletal rearrangement and inhibition of cell adhesion and spreading of HCC cells.

Transwell assays demonstrated that sh-MsrB1 reduced migration potential to 14.66% and 24.26% compared to the controls in LM3 and snu387 cells, respectively (Figure 5(a)).

### 3.9. Knockdown of MsrB1 Inhibits Cytoskeletal Rearrangement and Spreading

There were no noticeable differences in interior actin filament stress fiber formation in MsrB1-knockdown cells compared with control cells. However, regarding exterior actin filament stress fiber formation, ruffling and pseudopodium-induced cell migration were almost completely absent in MsrB1-knockdown cells compared with control cells. These pseudopodia function as drivers in HCC cells, and their absence may explain the reductions in HCC cell migration potential demonstrated by the transwell assay (Figures 5(b)).

### 3.10. Mechanism by Which MsrB1 Influences HCC Cell Proliferation

**Figure 6:**
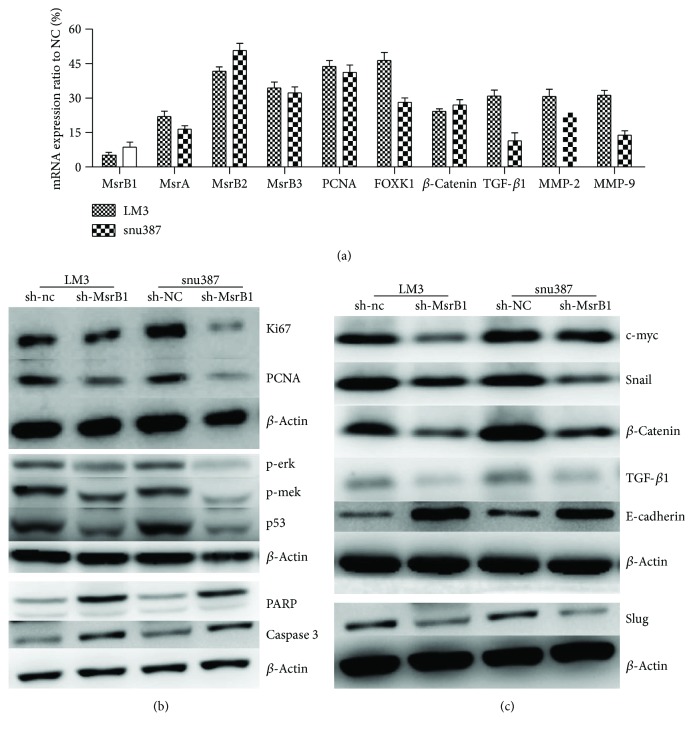
MsrB1 affects proliferation/migration of HCC cells by inhibition of the MAPK pathway, inducing apoptosis and inhibition of EMT. (a) The mRNA expression of MsrA/B2/B3, PCNA, FOXK1, *β*-catenin, TGF-*β*1, and MMP2/9 was regulated in MsrB1-knockdown cells. (b) The expression of protein phosphorylation of the key proteins of the MAPK pathway, namely, ERK, MEK, and level of p53, was inhibited, but activation and cleaved PARP and caspase 3 expression levels change reflective proliferation inhibition. (c) MsrB1 knockdown increased E-cadherin expression levels. However, MsrB1 knockdown decreased c-myc, Snail, TGF-*β*1, Slug, *β*-catenin, MMP-2, and MMP-9 expression levels, indicating that MsrB1 knockdown inhibited EMT.

Consistent with the results of the *in vitro* studies, our PCR results showed that knocking down MsrB1 induced downregulation of the related Msr genes MsrA, MsrB2, and MsrB3 (Figure 6(a)). We also observed that phosphorylation of the key proteins of the MAPK pathway, namely, ERK, MEK, and level of p53, was inhibited (Figure 6(b)). Ultimately, MsrB1 knockdown induced decreases in FOXK1 levels (a member of the forkhead protein family) and proliferating cell nuclear antigen (PCNA) and ki67 expression levels, changes which are reflective of proliferation inhibition (Figures 6(a) and 6(b)).

Furthermore, MsrB1 knockdown induced activation and cleaved PARP and caspase 3 expression levels, changes which are reflective of proliferation inhibition (Figure 6(b)).

### 3.11. Downregulation of MsrB1 in HCC Cells Inhibits EMT

EMT is essential for tumor invasion and migration in metastasis. To elucidate the mechanisms underlying this phenomenon, we examined the effects of MsrB1 knockdown on EMT by analyzing EMT-related factor expression in sh-MsrB1 cells (Figure 6(a)). MsrB1 knockdown increased E-cadherin expression levels. However, MsrB1 knockdown decreased c-myc, Snail, TGF-*β*1, Slug, *β*-catenin, MMP-2, and MMP-9 expression levels, indicating that MsrB1 knockdown inhibited EMT (Figures 6(a) and 6(c)).

We also stained *β*-catenin, p53, and Foxk1 in the HCC samples described above. Correlation analysis showed that both *β*-catenin and Foxk1 expression levels were positively correlated with MsrB1 expression levels (*P* = 0.0302, Table 3) (*P* = 0.000, Table 3). No correlation between MsrB1 expression and p53 expression was observed. We also analyzed the mRNA levels of the above proteins in the treated/untreated HCC cell lines and found that the *β*-catenin and Foxk1 mRNA levels were downregulated in the cell lines with MsrB1 interference (Figures 6(a)).

## 4. Discussion

HCC is the fifth cancer worldwide and is currently the third-leading cause of cancer-related death, as it accounts for half a million deaths each year. Over the years, there have been many advances in the therapeutic strategies used to treat HCC in its advanced or terminal stages. However, the overall prognosis of the disease has not improved. Although surgery is a suitable therapy for HCC in the terminal stages of the disease, novel therapeutic agents, prognosis-determining methods, and, in particular, biotherapies would clearly be of great benefit with respect to HCC treatment. Therefore, identifying biological markers that can contribute to HCC biotherapy is necessary.

Molecular oxygen is indispensable for the energy pathways that occur in various cellular compartments in aerobic organisms, but oxygen utilization is also associated with ROS generation [6]. Cancer cells exhibit increased levels of aerobic glycolysis (termed the Warburg effect) and high levels of ROS [1], whereas normal cells are dependent on oxidative phosphorylation. Accumulated ROS can damage various biomolecules, such as DNA, proteins, and lipids, and may contribute to the development of cancer [7, 8]. Methionine residues in proteins are susceptible to oxidation by ROS but can be repaired through MsrA- and MsrB-mediated reduction of resultant methionine sulfoxides. MsrB1 (selenoprotein R) is present in the cytosol and nucleus and exhibits the highest methionine-R-sulfoxide reductase activity levels in its family because of the presence of selenocysteine (Sec) in its active site. Given the characteristic metabolism associated with cancer cells, identifying an essential and highly specific target for cancer cells, an endeavor that is, comparatively, less important for normal cells, seems to be a feasible strategy for developing targeted biotherapies for HCC.

In the antioxidant enzymes induced by ROS, MSRs function in direct protein repair and indirect ROS scavenging. The vital cellular function of the Msr gene family entails protecting cells from oxidative damage by enzymatically reducing the oxidized sulfide groups of methionine residues in proteins from sulfoxide (-SO) back to sulfide, thus restoring normal protein function and reducing intracellular ROS levels [9]. The Msr family is a multigene family comprising the constitutively expressed proteins MsrA, MsrB1, MsrB2, MsrB3a, and MsrB3b, which participate in regenerating methionine from its oxidized form, Met(O). Of these three enzymes, MsrB1, a selenoprotein that contains a selenocysteine residue in place of the more conventional catalytic cysteine residue, is normally enriched relative to other MsrBs [10, 11] and displays high catalytic activity toward protein-based R-Met(O) and low activity toward free Met(O). However, a previous study found that MsrB1-knockout mice displayed normal development, indicating that MsrB1 is not an indispensable protein for tissue development [12] and behavior. In another study, MsrB1 genes were shown to be important for cell viability and for protecting lens cells from oxidative stress [13].

Researchers have been focused on the function of MsrB1 in *Drosophila* and human lens epithelial cells (HLEs). However, only a few studies have focused on the function of MsrB1 in tumor biology. In our study, MsrB1 was highly expressed in HCC tissues. MsrB1 expression was upregulated at the DNA, RNA, and protein levels in HCC tissues compared with paratumor tissues. The expression levels observed using IHC were correlated with the posthepatectomy prognoses of patients with HCC, and the results obtained via the above studies were also consistent with those obtained via database analysis.

Similar to the proteomic alterations observed in Msr-silenced HEK293 cells [14], the protein network alterations observed in the present study involved cellular processes, such as apoptosis, oxidative stress, necrosis, aging, DNA repair, protein degradation, and cytoskeletal formation, as well as metabolic processes, such as cellular respiration, fatty acid oxidation, and the tricarboxylic acid cycle. These results were confirmed by our observation that similar protein alterations were demonstrated via RNA-chip analysis of MsrB1-knockdown HCC cells. In this study, sh-MsrB1 cell proliferation was decreased compared with control cells, and apoptosis and cell cycle arrest, which are related to the process of cell proliferation, were upregulated by MsrB1 interference.

Tissue damage, including cell death, can result from the accumulation of high levels of free radicals in cells, which can cause oxidization and functional impairment directly or through signal transduction pathways, such as the c-Jun N-terminal kinase (JNK) and mitogen-activated protein kinase (MAPK) pathways [15]. According to the results of the RNA-chip analysis, MsrB1 knockdown affected many cellular processes. The predominant pathway affected was the MAPK pathway, and our Western blotting results indicated that Erk, MEK, and p53 were influenced by MsrB1. However, p53 expression was not affected by MsrB1 levels in HCC tissues. Therefore, we surmised that MsrB1 modified p53 expression. However, further studies are needed to support this idea.

Previous studies have demonstrated that MsrA overexpression protects lens cells against oxidative stress, whereas MsrA deletion renders these cells more vulnerable to oxidative stress and decreases cell viability in the absence of oxidative stress [16]. Here, we demonstrated that MsrB1 knockdown could decrease the expression of the other members of the Msr family. Therefore, we surmised that these enzymes function synergistically. Although our experiments did not distinguish among the individual activities of separate Msr enzymes acting on the same substrates, our PCR data demonstrated that all three Msr genes were expressed in HCC despite exhibiting different abundances in different sublocations. Antibodies for Msr proteins are not available; therefore, the corresponding protein expression levels could not be determined. Although we are confident that Msr protein expression levels paralleled the corresponding mRNA expression levels, we cannot rule out the possibility that differences between MsrB1 mRNA and protein expression exist. It is also unknown whether altering the expression levels of a specific enzyme in the Msr family can affect those of other members and/or whether a coregulation mechanism driving the expression of different Msr genes exists.

*In vitro*, HCC cell proliferation was affected by MsrB1 knockdown. Furthermore, the inhibition percentage was competitively decreased by sorafenib, a multikinase inhibitor. In addition, MsrB1 is an enzyme that distinctively assembles in the cytoplasm, not in the mitochondria. However, our results demonstrated that MsrB1 shRNAi can disrupt mitochondrial integrity, indicating that MsrB1 plays a role in mitochondrial function in HCC. This process is associated with apoptosis, and mitochondrial disruption or dysfunction can lead directly to cell death. Therefore, proliferation inhibition may be caused by the combined effects of cell death and apoptosis.

Regarding tumor migration and invasion, the cytoskeleton plays a major role in stimulating various processes that induce migration, including actin filament activation at the leading edge of the cell, and profilin-induced actin polymerization to propel the leading edge of the cell forward [17]. Our results indicated that there was no significant difference in interior stress fiber formation in MsrB1-knockdown cells compared with control cells. However, regarding exterior stress fiber formation, ruffling or pseudopodium-induced cell migration was almost completely absent in MsrB1-knockdown cells. The disappearance of driver-promoted cell movement disturbed tumor cell migration/invasion such that the transwell group displayed much slower migration/invasion than the control group.

Regarding the mechanism underlying the above phenomena, the morphological behaviors in question are related to EMT and processes associated with tumor metastasis. EMT is essential for tumor invasion and metastasis [18]. E-cadherin is the best-characterized molecular marker of EMT [19]. MsrB1 knockdown increased E-cadherin expression levels but decreased expression levels of *β*-catenin, Slug, and so on. This indicated that MsrB1 knockdown inhibited EMT. Many transcription factors, such as Snail, have been shown to directly or indirectly cause E-cadherin promoter activity repression [20]. We observed that *β*-catenin and Slug expression levels were decreased in MsrB1-knockdown HCC cells.

One of the most important causes of poor prognoses in cancer patients is tumor cell invasion of distal organs. The complex process of metastasis requires the integration of several events, including the dissociation of cells from the primary tumor in association with local remodeling and degradation of the ECM [21]. In our study, MMP-2 and MMP-9 expression levels were decreased in MsrB1-knockdown HCC cells.

In conclusion, ROS regulation is an important factor in tumor development, metastasis, and responses to anticancer therapies. ROS regulate many signaling pathways linked to tumorigenesis and metastasis, either directly or indirectly. Oxidative stress induction can lead to the preferential killing of cancer cells [3]. However, a previous study found that MsrB1-knockout mice display normal development, suggesting that MsrB1 is not an indispensable protein with respect to tissue development [12]. In this study, MsrB1-interferenced HCC cells showed decreased proliferation and metastasis, suggesting that this factor can be a biological target for tumor therapy.

Although the targets of MsrB1 in tumors have yet to be defined, it has been shown that MsrB1 is important for the maintenance of HCC cell viability and resistance to oxidative stress. These properties of MsrB1, coupled with the presence of the protein in HCC, may indicate that the protein is associated with the repair of proteins damaged by ROS and that loss of its normal function may contribute to disease progression.

We propose that targeting enhanced antioxidant defense mechanisms may be a useful strategy for specifically killing cancer cells while sparing normal cells [3]. Therefore, molecules that mediate MsrB1 expression and related processes may be the next important targets for future anticancer drug development studies.

## Figures and Tables

**Table 1:** The primers used for the RT-qPCR experiments.

Gene	Primer	Sequence
TGF-*β*1	Forward primer	GGCCAGATCCTGTCCAAGC
	Reverse primer	GTGGGTTTCCACCATTAGCAC
*β*-Catenin	Forward primer	CATCTACACAGTTTGATGCTGCT
	Reverse primer	GCAGTTTTGTCAGTTCAGGGA
MMP-2	Forward primer	TACAGGATCATTGGCTACACACC
	Reverse primer	GGTCACATCGCTCCAGACT
MMP-9	Forward primer	TGTACCGCTATGGTTACACTCG
	Reverse primer	GGCAGGGACAGTTGCTTCT
MsrA	Forward primer	GAGTGGTGTACCAGCCAGAAC
	Reverse primer	GGGTCGGGTCGTGATTCTC
MsrB2	Forward primer	CGGAGCAGTTCTACGTCACAA
	Reverse primer	CAGCACACGCAATGATACATTC
MsrB3	Forward primer	CGGTTCAGGTTGGCCTTCATT
	Reverse primer	GTGCATCCCATAGGAAAAGTCA
FOXK1	Forward primer	CAGTTACCGCTTTGTGCAGAA
	Reverse primer	CGGCTTTGACTCATCCTTGG
PCNA	Forward primer	CCTGCTGGGATATTAGCTCCA
	Reverse primer	CAGCGGTAGGTGTCGAAGC

**Table 2:** The relationship between MsrB1 expression levels and clinicopathological characteristics.

MsrB1 density			
Variable	High	Low	*P* value
In general			
Tumor tissue	80	32	
Sex			
Male	59	25	0.3
Female	14	3	
Age (years)			
≤50	22	15	*0.028*
>50	51	13	
Tumor size (cm)			
≤3	30	6	*0.04*
>3	45	25	
AFP (ng/ml)			
≤400	40	13	0.4251
>400	30	14	
HBsAg			
Positive	70	25	0.619
Negative	10	7	
Liver cirrhosis			
Yes	67	16	*0.04*
No	20	12	
BCLC			
0-B	77	27	*0.03*
C	1	3	
TNM stage (AJCC)			
I-II	72	29	0.96
III-IV	8	3	

MsrB1 expression levels were related to age (*P* = 0.028), tumor size (*P* = 0.04), liver cirrhosis (*P* = 0.04), and BCLC stage (*P* = 0.03).

**Table 3:** Correlation analysis of *β*-catenin and Foxk1 expression with MsrB1 expression levels.

MsrB1-related gene expression				
		MsrB1		
		Positive	Negative	*P* value
*β*-Catenin	Positive	66	24	
	Negative	18	16	*0.0302*
P53	Positive	31	13	
	Negative	59	21	0.6938
FOXK1	Positive	78	15	
	Negative	12	18	*0.00*

MsrB1 expression levels was related to *β*-catenin (*P* = 0.0302) and FOXK1 (*P* = 0.00), but there is no evidence to show the relation with the p53 level.

## Supplementary Materials

Supplementary 1 Figure S1: compared with patients with low expression, patients with high MsrB1 expression had worse survival in database analysis (*N* = 179, *P* = 0.201).

Supplementary 2 Figure S2: the predictive mechanism of the gene of MsrB1 in GO/KEGG enrichment. (A) The interference result of sh-MsrB1 on LM3 and snu387 cells. (B) Volcano plot of MsrB1 mRNA sequence expression levels was measured in sh-MsrB1 and sh-NC snu387 cells. (C) The predictive mechanism of the gene of MsrB1 in GO enrichment. (D) The predictive mechanism of the gene of MsrB1 in KEGG enrichment.

Supplementary 3 Figure S3: MsrB1 overexpression promotes proliferation and invasion in HCC cells. (A) MsrB1 overexpression promotes proliferation in huh7 cells through the MTS assay. (B) MsrB1 overexpression promotes proliferation in BEL7402 cells through the MTS assay. (C) MsrB1 overexpression promotes proliferation in huh7 cells with sorafenib through the MTS assay. (D) MsrB1 overexpression promotes proliferation in BEL7402 cells with sorafenib through the MTS assay. (E) Transwell assay-manifested MsrB1 overexpression promotes invasion of the huh7 cell. (F) Transwell assay-manifested MsrB1 overexpression promotes invasion of the BEL7402 cell. (G) Western blot indicated the different expression of MsrB1 in HCC cells with the pCMV-MsrB1 vector.
