# Supplementary material for: Methionine Sulfoxide Reductase B1 Regulates Hepatocellular Carcinoma Cell Proliferation and Invasion via the Mitogen-Activated Protein Kinase Pathway and Epithelial-Mesenchymal Transition
Source: Oxid Med Cell Longev. 2018 May 10;2018:5287971. doi: 10.1155/2018/5287971 (PMC5971335; doi:10.1155/2018/5287971)
Supplement: Supplementary 1 — Figure S1: compared with patients with low expression, patients with high MsrB1 expression had worse survival in database analysis (N = 179, P = 0.201). [file 5287971.f1.docx]

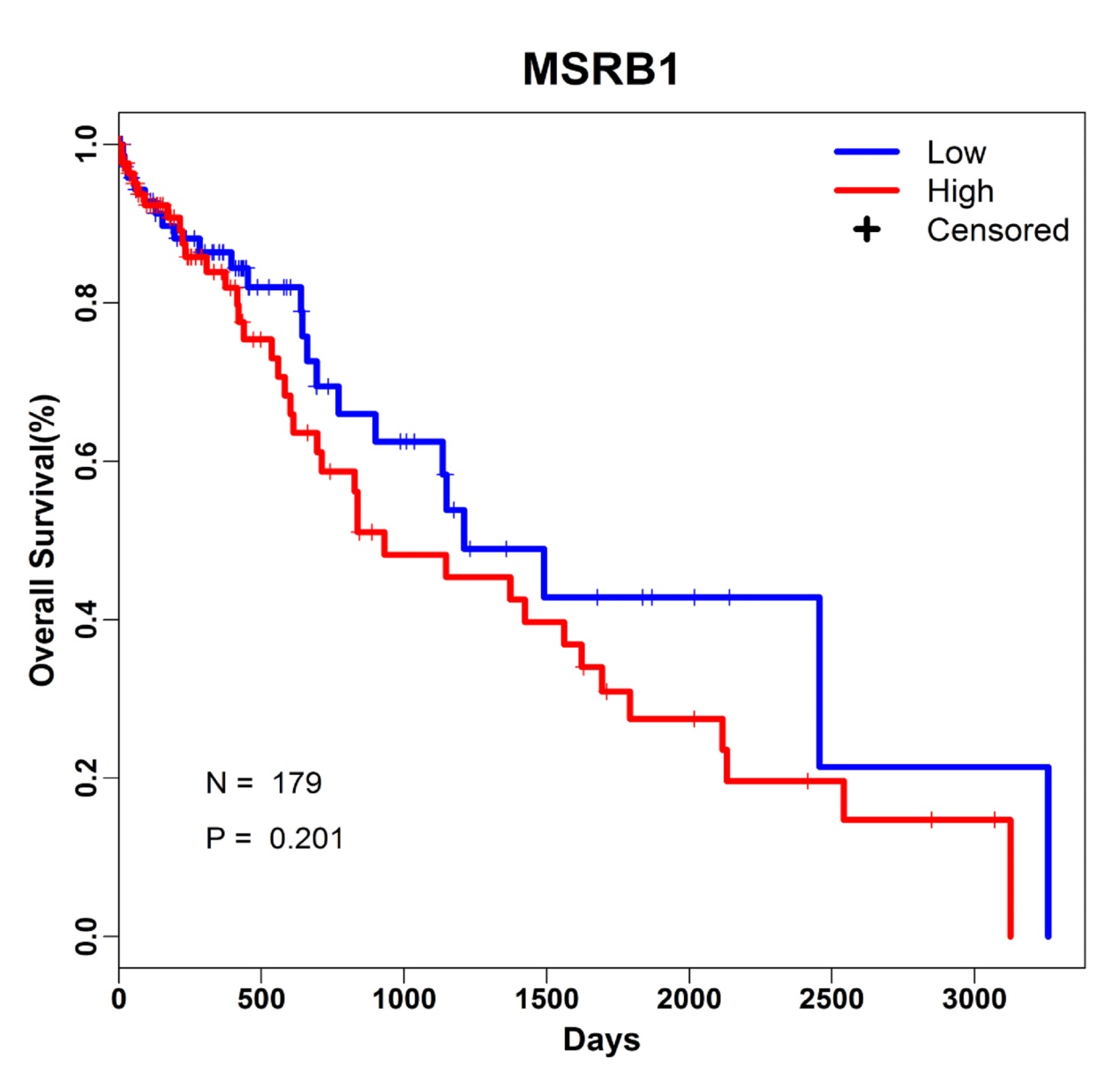


Figure S1 Compared with patients with low expression, patients with high MsrB1 expression had worse survival in database analysis(N=179, p=0.201).
