# Supplementary material for: Methionine Sulfoxide Reductase B1 Regulates Hepatocellular Carcinoma Cell Proliferation and Invasion via the Mitogen-Activated Protein Kinase Pathway and Epithelial-Mesenchymal Transition
Source: Oxid Med Cell Longev. 2018 May 10;2018:5287971. doi: 10.1155/2018/5287971 (PMC5971335; doi:10.1155/2018/5287971)
Supplement: Supplementary 2 — Figure S2: the predictive mechanism of the gene of MsrB1 in GO/KEGG enrichment. (A) The interference result of sh-MsrB1 on LM3 and snu387 cells. (B) Volcano plot of MsrB1 mRNA sequence expression levels was measured in sh-MsrB1 and sh-NC snu387 cells. (C) The predictive mechanism of the gene of MsrB1 in GO enrichment. (D) The predictive mechanism of the gene of MsrB1 in KEGG enrichment. [file 5287971.f2.docx]

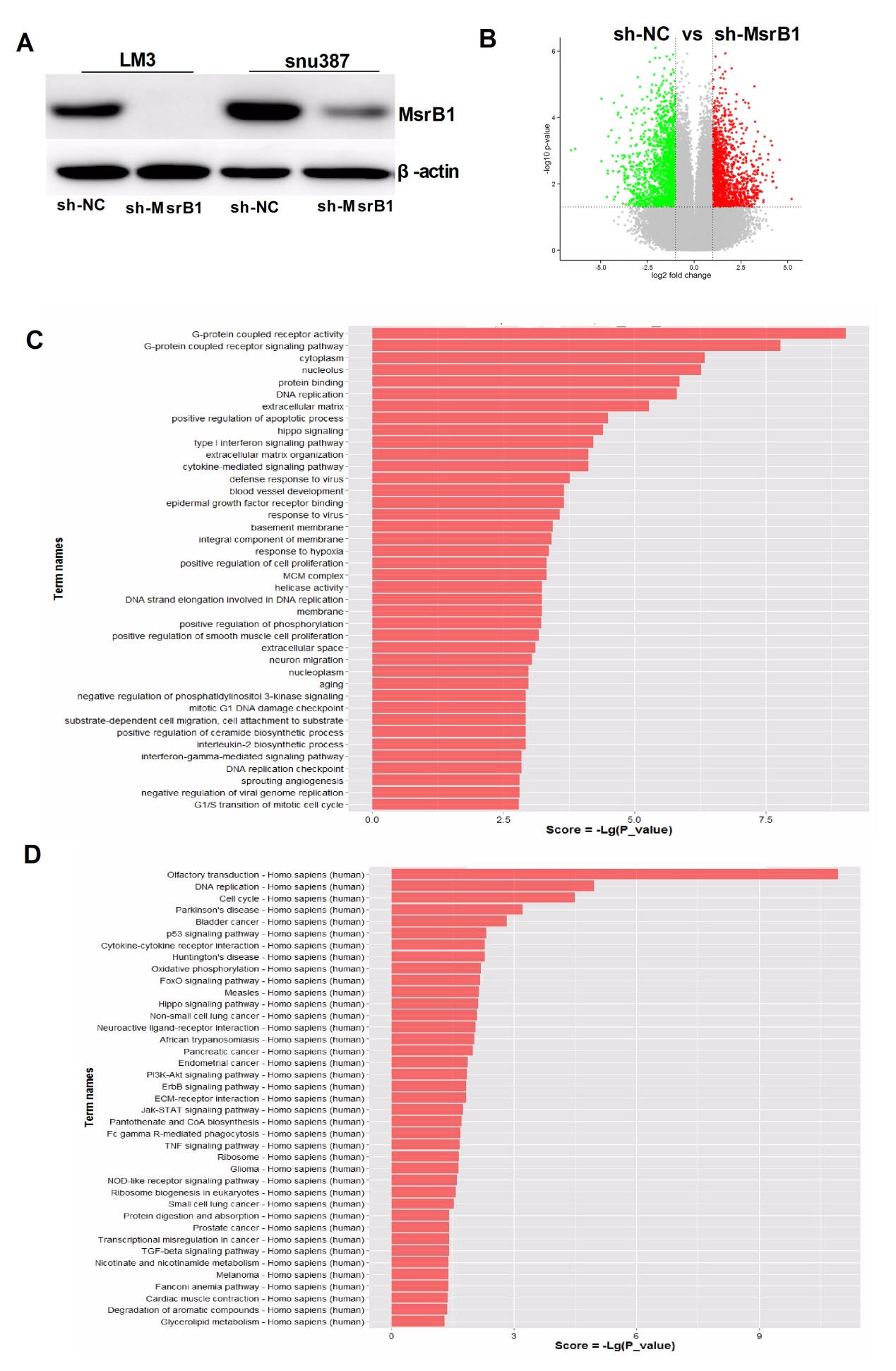


Figure S2 the predictive mechanism of gene of MsrB1 in GO/KEGG enrichment. A. the interference result of sh-MsrB1 on LM3 and snu387 cells. B. Volcano plot of MsrB1 mRNA sequence expression levels were measured in sh-MsrB1 and sh-NC snu387 cells .C. the predictive mechanism of gene of MsrB1 in GO enrichment. D. the predictive mechanism of gene of MsrB1 in KEGG enrichment.
