# Supplementary material for: Methionine Sulfoxide Reductase B1 Regulates Hepatocellular Carcinoma Cell Proliferation and Invasion via the Mitogen-Activated Protein Kinase Pathway and Epithelial-Mesenchymal Transition
Source: Oxid Med Cell Longev. 2018 May 10;2018:5287971. doi: 10.1155/2018/5287971 (PMC5971335; doi:10.1155/2018/5287971)
Supplement: Supplementary 3 — Figure S3: MsrB1 overexpression promotes proliferation and invasion in HCC cells. (A) MsrB1 overexpression promotes proliferation in huh7 cells through the MTS assay. (B) MsrB1 overexpression promotes proliferation in BEL7402 cells through the MTS assay. (C) MsrB1 overexpression promotes proliferation in huh7 cells with sorafenib through the MTS assay. (D) MsrB1 overexpression promotes proliferation in BEL7402 cells with sorafenib through the MTS assay. (E) Transwell assay-manifested MsrB1 overexpression promotes invasion of the huh7 cell. (F) Transwell assay-manifested MsrB1 overexpression promotes invasion of the BEL7402 cell. (G) Western blot indicated the different expression of MsrB1 in HCC cells with the pCMV-MsrB1 vector. [file 5287971.f3.docx]

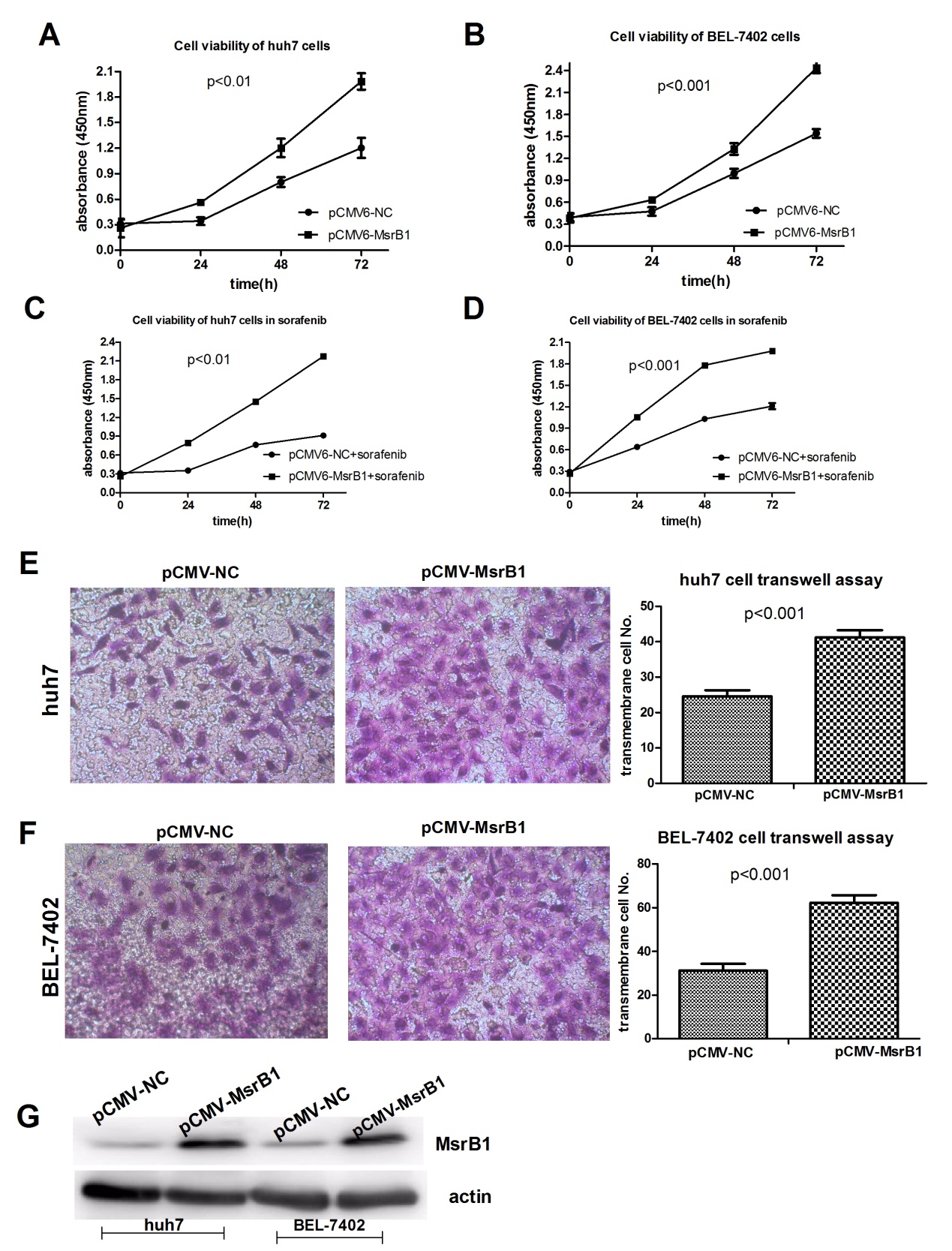


Figure S3. MsrB1 overexpression promotes proliferation and invision in HCC cells. A. MsrB1 overexpression promotes proliferation in huh7 cells through MTS assay. B. MsrB1 overexpression promotes proliferation in BEL7402 cells through MTS assay. C. MsrB1 overexpression promotes proliferation in huh7 cells with sorafenib through MTS assay. D. MsrB1 overexpression promotes proliferation in BEL7402 cells with sorafenib through MTS assay. E.Transwell assaymanifested MsrB1 overexpression promotes invision of huh7 cell. F. Transwell assaymanifested MsrB1 overexpression promotes invision of BEL7402 cell. G. western blot indicated the different expression of MsrB1 in HCC cells with pCMV-MsrB1 vector.
